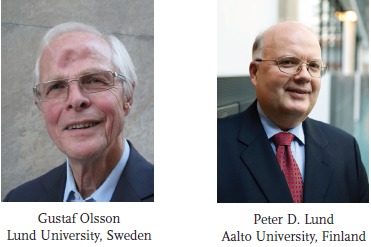# Water and Energy – Interconnections and Conflicts

**DOI:** 10.1002/gch2.201700056

**Published:** 2017-08-14

**Authors:** Gustaf Olsson, Peter D. Lund

**Affiliations:** ^1^ Lund University Sweden; ^2^ Aalto University Finland

Water and energy are key global challenges, and they are strongly interlinked. They affect everyday life. Energy extraction and generation requires water, and water supply depends on energy. This coupling has enormous significance for all humans. For almost all energy production water is a critical ingredient, still not sufficiently recognized. Water is needed for fossil fuel extraction, transport, and processing, for power generation as well as for irrigation of biofuel feedstock. Energy is needed for the complete urban water cycle, from water abstraction to water treatment as well as for used water collection and treatment. A large fraction of the required energy goes for pumping, which will increase in the future, because growing water scarcity will demand longer water transports in pipes and channels. Energy can also be produced in the form of biogas as a by‐product from organic sludge treatment.

The United Nations has presented 17 sustainable development goals (SDG) to transform our world. Two of these (SDG 6 and 7) are directly related to our topic: clean water and sanitation, affordable and clean energy for all. Moreover, without adequate access to water and energy we will not be able to eliminate hunger (SDG2). Both water and energy are very closely coupled to climate change and climate action (SDG13). Life below water (SDG14) and life on land (SDG15) cannot exist without clean water.

In 2016 the World Economic Forum conducted a ranking of global risks.^1^ Three out of the top‐five risks are concerned with energy (a failure of climate change mitigation and adaptation, or a severe energy price shock) or water (water crises).

Peter Gleick^2^ raised the issue of the increasing energy need and water scarcity already in 1994, but the water‐energy conflicts were not fully recognized at that time. Most of the energy industry still took water availability for granted. Dr. Allan Hoffman, then a senior analyst at the U.S. Department of Energy in Washington DC, became one of the pioneers who, before the turn of the century, recognized the strong coupling between energy and water.^3^, ^4^ In a commentary to this Special Issue (see AdvancedScienceNews.com) he describes his early experiences on the efforts to raise the awareness of the water‐energy interconnections. He actually was the first one to phrase the term water‐energy nexus that has become the buzzword for these coup­lings. Allan Hoffman has also been instrumental in the work resulting in the U.S. DOE report^5^ that contains a comprehensive description of many of the problems that appear when water availability for energy production is no longer guaranteed.

The paper by Webber^6^ was an eye opener for many. The interdependencies of water and energy have gradually been recognized by policy makers, the United Nations,^7^ the World Bank,^8^ businesses,^9^ and many other stakeholders, such as the Union of Concerned Scientists.^10^ The International Energy Agency (IEA) paid special attention to the water requirements in the energy sector in its World Energy Outlook^11^ in 2012. In 2015, the IEA considered how water scarcity influences the choice of cooling technology in coal‐fired power plants in India and China.^12^ In 2016, a whole chapter of the World Energy Outlook was devoted to the water‐energy nexus.^13^ The European Water Supply and Sanitation Technology Platform (WssTP) documented research needs for water and energy.^14^ The IPCC Assessment Reports contain a lot of information on the water‐energy nexus. The water‐energy interconnections are also documented in a book.^15^ An excellent summary of the energy‐water‐food nexus was recently published by the Danish Technical University.^16^

The close relation between energy and water, and its importance as a global challenge was the motivation to produce a Special Issue on the Water‐Energy Nexus, with five papers representing different perspectives to the challenge. This Special Issue aims at describing the water‐energy interconnections and their global impact for an audience interested in global challenges. The primary ambition is to raise the awareness of these challenges. It is our hope and aspiration to find approaches and methodologies to deal with the water‐energy nexus at different scales.

It is most apparent that hydropower production is determined by water availability. However, water is also lost due to evaporation in hydropower reservoirs. This problem is discussed in the paper by Tor Haakon Bakken, Ånund Killingtveit, and Knut Alfredsen from the Norwegian University of Science and Technology, Trondheim. Hydropower has also an important role to play when increasing the share of non‐traditional renewable electricity, such as wind and solar PV. The new renewables need negligible amounts of water and will have a profound impact on water supply, as described in the paper by Lawrence Jones from Monash University, Melbourne (Australia) and Gustaf Olsson, Lund University (Sweden).

The water‐energy nexus is very well recognized in China and tremendous efforts are made to solve the water scarcity problem created by energy generation. The energy needs for water supply and wastewater treatment in urban areas of China are explained by Kate Smith and Shuming Liu from Tsinghua University, Beijing.

Climate change has a very strong connection to energy and water on several levels. D. Dennis Konadu and Richard A. Fenner from the University of Cambridge (UK) describe how the UK government has proposed different low‐carbon energy systems and estimate the water requirement for the deployment of the proposed energy technology mix.

Primary energy extraction and production has led to a whole spectrum of environmental challenges. In the paper by Nenibarini Zabbey, University of Port Harcourt (Nigeria) and Gustaf Olsson the conflicts between oil exploration and water are demonstrated.

Given all the interrelationships between water and energy it is apparent that the subject has to be approached in an integrated way. Still the delivery chains of water and energy are mostly managed in ‘silos’, where the silos not only represent different professions and sectors but also different institutions. It is apparent that our infrastructures of energy and water have to be designed and operated in a more integrated way. This challenge goes all the way up to government agencies and ministries. The Malaysian Government may serve as a good example with their *Ministry of Energy, Green Technology and Water*. The collaboration between the stakeholders has also to be strengthened. The ultimate goal should be to reach the UN Sustainable Development Goals.

We hope that this issue will inspire further work in an area that is vital for our future.